# Supplementary material for: Sleep and Economic Status Are Linked to Daily Life Stress in African-Born Blacks Living in America
Source: Int J Environ Res Public Health. 2022 Feb 23;19(5):2562. doi: 10.3390/ijerph19052562 (PMC8909882; doi:10.3390/ijerph19052562)
Supplement: Supplementary file 1 [file ijerph-19-02562-s001.zip › ijerph-1514148-supplementary.pdf]

Supplement Table S1. The 10 Questions and Scoring System of the Perceived Stress Scale[1].

Questions	Scoring System[1]
1. **In the past month**, how often have you been upset because of something that happened unexpectedly?	A
2. **In the past month**, how often have you felt unable to control the important things in your life?	A
3. **In the past month**, how often have you felt nervous or stressed?	A
4. **In the past month**, how often have you felt confident about your ability to handle personal problems?	B
5. **In the past month**, how often have you felt that things were going your way?	B
6. **In the past month**, how often have you found that you could not cope with all the things you had to do?	A
7. **In the past month**, how often have you been able to control irritations in your life?	B
8. **In the past month**, how often have you felt that you were on top of things?	B
9. **In the past month**, how often have you been angry because of things that had happened that were out of your control?	A
10. **In the past month**, how often have you felt that difficulties were piling up so high that you could not overcome them?	A

[1]Scoring System (Reference 5):

A (Negative): 0=Never, 1=Almost Never, 2=Sometimes, 3=Fairly Often, 4=Very Often

B (Positive): 0=Very Often, 1=Fairly Often, 2=Sometimes, 3=Almost Never, 4=Never
